# Neurodegeneration in Schizophrenia: Evidence from *In Vivo* Neuroimaging Studies

**DOI:** 10.1100/tsw.2007.47

**Published:** 2007-02-02

**Authors:** John G. Csernansky

**Affiliations:** Departments of Psychiatry and Anatomy and Neurobiology, Washington University School of Medicine, St. Louis, MO 63110, USA

**Keywords:** schizophrenia, neuroimaging, neurodegeneration, glutamate, apoptosis

## Abstract

Although schizophrenia is primarily considered to be a neurodevelopmental disorder, there is a growing consensus that the disorder may also involve neurodegeneration. Recent research using non-invasive neuroimaging techniques, such as magnetic resonance imaging, suggests that some patients with schizophrenia show progressive losses of gray matter in the frontal and temporal lobes of the brain. The cellular mechanisms responsible for such gray matter losses are unknown, but have been hypothesized to involve abnormal increases in apoptosis.

## INTRODUCTION

Schizophrenia is a potentially disabling disorder because of its capacity to destroy our normal capacities for perception, reasoning, and interpersonal relationships. Neuroanatomical abnormalities have been observed in patients with schizophrenia, and are thought to represent the structural substrate for the disorder's symptoms and signs. While the neuroanatomical abnormalities observed in schizophrenia may originate from a neurodevelopmental defect[111], there is growing evidence that the magnitude and pattern of these abnormalities progress over time[62]. The combination of neurodevelopmental and neurodegenerative processes within a single disease entity is a challenging but plausible possibility[18].

## Patterns of Neuroanatomical Abnormalities in Schizophrenia

In 1976, Johnstone and colleagues used computerized tomography (CT) to demonstrate that schizophrenic patients have enlarged lateral cerebral ventricle[58]. Since that time, much effort has been devoted to characterizing the tissue losses that can account for this expansion of the ventricular system[21,113]. Reports of decreases in the volume of the entire temporal lobe[100] were followed by reports of hippocampal volume reduction in individuals with schizophrenia by numerous groups [6,88,7,85,9,70,45,19,23]. However, the degree of hippocampal volume reduction associated with schizophrenia tends to be small (~5 %) (79,116]. Similarly, reductions in the volume of the parahippocampal gyrus were reported[88,61,73]. Again, however, a meta-analysis of such studies found the degree of volume reduction to be small (~5%)[116].

There have also been several reports of reductions in the gray matter volume of cortical structures in individuals with schizophrenia. Early studies using magnetic resonance imaging reported reductions in the total volume of the frontal lobe[2], as well as the gray[119,43,86]and white matter volume of the entire frontal lobe[7]. More recently, there have been reports of volume reductions in particular cortical subregions[10,16]). Voxel-based studies of cortical structure in schizophrenia also suggest that gray matter volume loss is not homogeneous in schizophrena. To date, gray matter volume losses have been more prominent in fronto-temporal cortical region[65,24].

The thalamus has dense reciprocal connections with the cerebral cortex, and volume reductions of the thalamic complex have also been reported in individuals with schizophrenia (see [64] for meta-analysis). Of course, the thalamus is not a homogeneous structure and individual nuclei project topographically to specific regions of the cerebral cortex[5]. In keeping with the heterogeneity of cortical findings, post-mortem and neuroimaging studies of individual thalamic nuclei in individuals with schizophrenia suggest specific involvement of the anterior nucleus, mediodorsal nucleus and pulvinar. Notably, these components of the thalamic complex all project to fronto-temporal regions of the cortex[81,11,12]. Using methods that define abnormalities of the surface of the thalamic complex, deformations in the anterior and posterior extremes of the structure have also been reported, which is in keeping with localized volume loss in the anterior, anterolateral, dorsomedial nuclei and pulvinar[22].

Not all investigators have been able to confirm these findings. For example, no significant differences in corticolimbic brain region volumes, including the hippocampus and amygdala, in schizophrenia patients compared to healthy controls were found[102],. Similarly, no evidence of hippocampal volume reduction in schizophrenia subjects after correction for differences in total brain volume were found[120,72]. Bogerts et al[5], found decreases in amygdala/hippocampus volumes in schizophrenia subjects, but only in males. Also, Kawazaki, et al[61], found no differences in total temporal lobe volumes or hippocampal volumes, and [32,31], found no differences in total temporal lobe volumes between schizophrenia subjects and controls. Failure to replicate findings of neuroanatomical abnormalities in schizophrenia is unfortunately common, probably because of measurement error and the biological heterogeneity of patient populations[83].

While the cellular basis of gray matter volume losses in schizophrenia is not yet known, it appears that it is more likely due to the loss or disorganization of neuronal processes than the loss of neuronal cell bodies. Several post-mortem studies have reported abnormal neuronal organization within corticolimbic structures in schizophreni[38,54,15,1]. Using MR imaging, Kikinis, et a[63], studied gyral folding on the lateral surface of the temporal lobe and reported that the schizophrenia subjects demonstrated vertical sulcal patterns more often than healthy controls. Toga and colleagues have developed statistical variability maps for cortical sulci in normal individual[103,104,33], have begun to apply these methods to discriminate groups of schizophrenia and control subjects. Several investigators have also reported distortions of normal patterns of cortical asymmetries in schizophrenia[3,69,59,6, 105]. Also, Shenton, et a[88], reported hippocampal volume reductions only on the left side, perhaps suggesting an exaggeration of the normative L<R pattern of hippocampal asymmetry (see also [109,23]. Post-mortem studies also suggest there are more prominent abnormalities in the left temporal lobes of schizophrenia subjects, including temporal horn enlargement[8,17,48] and neuronal heterotopia[54].

## Evidence for the Progression of Neuroanatomical Abnormalities in Schizophrenia

Early studies of brain structure using pneumoencephalography suggested that ventricular enlargement might be progressive in schizophrenia[47, see also [29,30] for review]. However, with the advent of computerized tomography and magnetic resonance imaging, many investigators formed the opinion that neuroanatomical abnormalities in schizophrenia subjects were unchanging, and therefore of neurodevelopmental origin (see[111]. Woods and Wolf[114] were among the first to report evidence from *in vivo* neuroimaging studies that ventricular enlargement was progressive in schizophrenia, although they supported this claim by comparing the degree of ventricular enlargement in first-episode and chronic schizophrenia patients rather than examining longitudinal data.

The question of whether neuroanatomical abnormalities observed in schizophrenia are static or progressive is not yet fully resolved. While some investigators suggest that ventricular enlargement and gray matter volume losses are progressive over periods of one to five years in schizophrenia subjects[25,77,115,51], others suggest that these same structural measures are highly stable over similar time periods[53,78,108]. Differences in scanning and segmentation methods could account, at least in part, for disparities in these findings. For example, magnetic resonance scanners undergo a certain amount of “drift” over time, and the degree to which this drift can be accounted for when making longitudinal measurements would influence the degree to which time-dependent changes are detected. Also, errors inherent in many manual segmentation methods across manual operators and even within operators over time can be large relative to changes associated with schizophrenia[46].

To examine the possibility that progressive neuroanatomical changes might occur only during certain phases of schizophrenia, especially early in the course of illness, studies of individuals with schizophrenia in their first episode of illness have been undertaken. However, these studies also provide evidence both in favor of[28,44,49,67,71,13,50,40] and against[27,90,57,107] the claim that there is progressive gray matter losses in schizophrenia. Even in studies reporting progressive neuroanatomical abnormalities, there has been variation in the brain structures involved. For example, Hirayasu, et al[49], found evidence of gray matter volume losses in the left superior temporal gyrus, while Gur, et al.[44] found evidence of progressive volume losses in the frontal lobes. Lieberman, et al[67], reported increases in ventricular volumes, but no reductions in cortical or hippocampal gray matter volumes. Finally, Gur, et al[44], reported evidence of progressive temporal lobe volume losses in both schizophrenia and healthy control subjects. More recent studies of first episode schizophrenia subjects have been arguably more consistent[13,50,60,40]. Across these studies, there has been more consistent evidence of progressive gray matter losses in fronto-temporal cortical regions.

Recent studies of individuals with “prodromal” schizophrenia have suggested that there may be relatively rapid changes in neuroanatomical structure early in the course of illness [62]. Pantelis and colleagues[82] have conducted a series of remarkable studies of brain structure in subjects with prodromal symptoms of schizophrenia, some of whom go on to convert to fulfilling full criteria for the diagnosis of schizophrenia. Using voxel-based methods, they found that subjects who progressed from a prodromal state to full-blown psychosis showed gray matter losses in the parahippocampal, fusiform, orbitofrontal and cingulate gyri over approximately 12 months as compared to prodromal subjects who did not progress[82]. Cross-sectional studies of the cingulate gyrus in prodromal subjects suggest that abnormalities of the cingulate gyrus may also predict clinical progression[112,117]. However, it is still unclear as to whether hippocampal volume losses occur during the progression from prodrome to psychosis[112,106].

## Possible Mechanisms Underlying Neurodegeneration in Schizophrenia

Initial attempts to characterize the “neurodegenerative” process in schizophrenia focused on efforts to detect gliosis in the brains of individuals with schizophrenia[14]. However, little evidence of a gliotic reaction typical of a neurodegenerative process has been found. Next, investigators sought evidence of occult Alzheimer’s disease (AD) pathology. However, careful study of post-mortem material[4], even from subjects with gross dementia[84], ruled out the presence of AD-like neuropathology. Also, genetic markers related to AD (i.e., apoE4) do not appear to be associated with schizophrenia[41].

Several alternate mechanisms of neuronal injury are now being investigated in relationship to the pathogenesis of schizophrenia. Olney and colleagues have suggested that a developmental deficit of NMDA receptor-bearing GABAergic interneurons would place an individual at increased risk for excitotoxic neuronal injury later in life[80]. Excitotoxicity (i.e., neurodegeneration via the overactivity of excitatory neurotransmission) is an attractive mechanism to explain neuronal injury in schizophrenia because it could be initiated and/or maintained through the action of neurotransmitter systems, such as the monoamines, that have long been implicated in schizophrenia[39]. Also, glucocorticoid hormones[87], triggered by environmental stressors, including those (e.g., famine) associated with an increased risk for schizophrenia[101], have been implicated.

Another attractive theory to explain neuronal injury in schizophrenia is the inappropriate activity of apoptosis[42], a process normally associated with the elimination of redundant neurons during development[34]. In rats, brief perinatal exposure to sublethal doses of the excitotoxin, kainic acid, has been shown to trigger ongoing apoptosis-related neuronal losses in corticolimbic structures through pubescence[[75,52]. Among the neurons lost through this process are NMDA receptor-bearing GABAergic interneurons[36]. Also, neurogenesis is stimulated within the subgranular zone of the dentate gyrus and newly-generated cells generated within this area migrate toward brain regions with the greatest density of apoptotic neurons[35] – possibly in an attempt to replace damaged or dying neurons. Thus, an early excitotoxic injury could increase the vulnerability of limbic structures to ongoing apoptosis later in life and promote unusual patterns of neuroanatomical reorganization.

Basic studies of CNS development suggest that different populations of neurons and neuron precursor cells alter their expression of apoptosis-related genes at different times during neurodevelopment[34]. The expression of genes related to apoptosis versus neuronal survival (e.g., Bcl2 and Bax) decreases in most brain regions after birth, but not in brain areas related to learning and memory, such as the structures of the medial temporal lobe[74]. Possibly, in such brain regions, increased expression of Bcl2 is needed to support processes such as adult neurogenesis[34]. Also, components of molecular pathways related to neuronal differentiation, the growth of neuronal processes and neuroprotection, such as Notch[66] and PACAP[89], are densely expressed in fronto-temporal cortical structures. However, as a counterpoint to these factors, enzymes involved in the apoptosis cascade, such as capsase3, are also densely expressed in the same structures[118]. Finally, the expression of BDNF is especially high in the hippocampus and parahippocampal gyrus[76]. Thus, the integrity of fronto-temporal structures may be especially dependent on competing molecular processes that determine neuronal survival or degeneration. The regional localization of these processes provides a plausible basis for observations that fronto-temporal cortical structures show the greatest degree of structural change over time in individuals with schizophrenia.

Recent studies of allelic variation and gene expression in subjects with schizophrenia provide preliminary support for the idea that the regulation of neuronal survival mechanisms is abnormal in schizophrenia. Allelic variation in the Notch4 gene has been linked to frontal lobe gray volume decreases in subjects with schizophrenia[110]. Also, Jarskog, et al[55,56], reported post-mortem decreases in Bcl2 expression and increases in Bax expression in the temporal cortex of subjects with schizophrenia. Interestingly, this group has also proposed that dysregulated apoptosis may underlie neuropil loss, currently the most likely basis for gray matter volume loss in subjects with schizophrenia[42].

## Can Treatment Modify the "Neurodegeneration" Associated with Schizophrenia?

The results of a recent study of first episode schizophrenia patients randomly assigned long-term treatment with olanzapine or haloperidol suggest that antipsychotic drug treatment may affect the progression of neuroanatomical abnormalities associated with schizophrenia[68]. In this study, haloperidol treatment was associated with a greater rate of cortical gray matter loss than olanzapine, suggesting that the olanzapine might slow the progressive loss of cortical gray matter in subjects with schizophrenia (or that haloperidol may aggravate it). In non-human primate[37], reported that both haloperidol and olanzapine administration was associated with decreases in brain volume[37]. Recently, using the rat model of kainic acid-induced apoptosis described above, our group found that olanzapine, but not haloperidol, reduced apoptosis-related neuronal loss in the hippocampus and cortex[20]

Unfortunately, results from a European study of changes in brain structure during treatment with typical versus atypical antipsychotic drugs in first episode patients (i.e., the AESOP study) suggest relatively few differences in the rate of cortical gray matter loss[26]. Curiously, McCormick, et al (2005)[121], reported that treatment with typical antipsychotic drugs was associated with *increases* in anterior cingulate volume, while treatment with atypical antipsychotic drugs was associated with *decreases* in anterior cingulate volume. Thus, to date, there is little consensus as to whether existing treatments for schizophrenia can alter the underlying neurobiological pathogenesis of the disease.

In summary, there is growing evidence that the pathogenesis of schizophrenia includes a neurodegenerative component. While current drug treatments for schizophrenia are reasonably adequate for controlling the day-to-day symptoms of schizophrenia, they do not appear to alter the long-term clinical and biological outcomes of the disease. As the features of the "neurodegeneration" associated with schizophrenia become better understood, new targets for treatment intervention may be identified.
